# Research progress on the role of macrophages in acne and regulation by natural plant products

**DOI:** 10.3389/fimmu.2024.1383263

**Published:** 2024-04-26

**Authors:** Dan Zhao, Yun Wang, Shuhui Wu, Xiaotian Ji, Ke Gong, Huie Zheng, Mingfang Zhu

**Affiliations:** ^1^ Department of Dermatology, The Second Affiliated Hospital of Hunan University of Chinese Medicine, Changsha, China; ^2^ Department of Traditional Chinese Medicine, Cangzhou Central Hospital, Cangzhou, China

**Keywords:** macrophages, acne vulgaris, inflammatory response, sebum metabolism, natural plant products

## Abstract

Acne vulgaris is one of the most common skin diseases. The current understanding of acne primarily revolves around inflammatory responses, sebum metabolism disorders, aberrant hormone and receptor expression, colonization by *Cutibacterium acnes*, and abnormal keratinization of follicular sebaceous glands. Although the precise mechanism of action remains incompletely understood, it is plausible that macrophages exert an influence on these pathological features. Macrophages, as a constituent of the human innate immune system, typically manifest distinct phenotypes across various diseases. It has been observed that the polarization of macrophages toward the M1 phenotype plays a pivotal role in the pathogenesis of acne. In recent years, extensive research on acne has revealed an increasing number of natural remedies exhibiting therapeutic efficacy through the modulation of macrophage polarization. This review investigates the role of cutaneous macrophages, elucidates their potential significance in the pathogenesis of acne, a prevalent chronic inflammatory skin disorder, and explores the therapeutic mechanisms of natural plant products targeting macrophages. Despite these insights, the precise role of macrophages in the pathogenesis of acne remains poorly elucidated. Subsequent investigations in this domain will further illuminate the pathogenesis of acne and potentially offer guidance for identifying novel therapeutic targets for this condition.

## 1 Introduction

Acne is a prevalent chronic inflammatory skin disease affecting the hair follicles and sebaceous glands, with skin lesions primarily appearing on the face and chest back. The clinical manifestations encompass a diverse range of acneiform lesions, including comedones, papules, pustules, nodules, and cysts. These are often accompanied by excessive sebum secretion (1–3). Acne, affecting 9.4% of the global population, ranks as the eighth most prevalent disease worldwide. Despite its clinical commonality, post-acne erythema and scarring contribute significantly to psychological disorders among young individuals, thereby elevating the risk of mood disorders and psychiatric comorbidity (4–7). Consequently, acne has emerged as a significant societal concern. Currently, the internationally recommended pharmacological treatments for acne encompass topical retinoids, benzoyl peroxide, antibiotic ointments, as well as oral antibiotics and anti-androgen medications (8–10). Despite their efficacy, these existing therapeutic strategies often give rise to adverse effects such as cutaneous irritation, dryness, and disturbances in the skin’s microecology due to topical agents. Additionally, concerns regarding the emergence of antibiotic-resistant bacteria have also been raised (1). The management of acne is increasingly challenging, thus necessitating an urgent exploration of novel targets and mechanisms in acne research.

The pathogenesis of acne involves inflammatory responses, hypersecretion of sebum, abnormal keratinization of the sebaceous glands in hair follicles, colonization by *Cutibacterium acnes*(*C. acnes*), and dysregulated androgen secretion (11–15). The inflammatory response is observed throughout the entire course of acne (16). Increasing evidence suggests that the inflammatory reaction in acne is associated with abnormal sebum metabolism, colonization of *C. acnes*, and abnormal androgen levels. Macrophages are essential components of the inflammatory response and, as integral members of the immune effector cells, also contribute significantly to maintaining skin homeostasis. Hence, it is imperative to investigate the role of macrophages in the pathogenesis of acne. In this review, we initially introduced the pivotal role of macrophages in skin physiology, elucidated their intricate associations with sebum metabolism, androgen regulation abnormalities, and *C. acnes* colonization, followed by a comprehensive summary of natural compounds that modulate the phenotypic alterations of acne-associated macrophages. This review further elucidates the correlation between macrophages and acne pathogenesis, as well as contributes to the development of potential therapeutic agents.

## 2 Role of macrophages in the skin

Macrophages are derived from hematopoietic stem cell-derived monocytes in the bone marrow (17), or originate from all tissues during embryonic development through the yolk sac and fetal liver, subsequently establishing themselves as resident cells within the tissues (18, 19). Macrophage populations exhibit significant heterogeneity across various tissues and perform important physiological functions specific to the tissues in which they reside. In general, resident macrophages maintain tissue homeostasis, while monocyte-derived macrophages primarily contribute to host defense and pathological signaling (20, 21). Additionally, macrophages exhibit significant plasticity and undergo polarization in response to environmental changes within different tissues, giving rise to distinct subtypes of macrophages (22).

Traditionally, macrophages can be polarized into two distinct phenotypes, namely classically activated M1 macrophages and alternatively activated M2 macrophages (23). M1 macrophages are stimulated by lipopolysaccharide (LPS) and interferon-γ (IFN-γ) (24, 25) to elicit a pro-inflammatory response and secrete various pro-inflammatory factors, including interleukin (IL)-1β, IL-6, IL-12, IL-23, inducible nitric oxide synthase (iNOS), monocyte chemotactic protein-1 (MCP-1), and tumor necrosis factor-alpha (TNF-α) (18, 26). Activated M1 macrophages promote Th1 cell-mediated immune responses, thereby safeguarding the host (27, 28). Additionally, they generate reactive oxygen species (ROS) and active nitrogen intermediates (29), which effectively eliminate pathogens. However, these mechanisms can also lead to collateral tissue damage and excessive inflammation, consequently impeding tissue regeneration and wound healing processes (30, 31). During the process of skin pathology, M1 macrophages play a crucial role in eliminating pathogenic microorganisms. However, sustained polarization toward the M1 phenotype can perpetuate a highly inflammatory state within the lesions, impeding optimal wound tissue healing (32–34).

**Figure 1:**
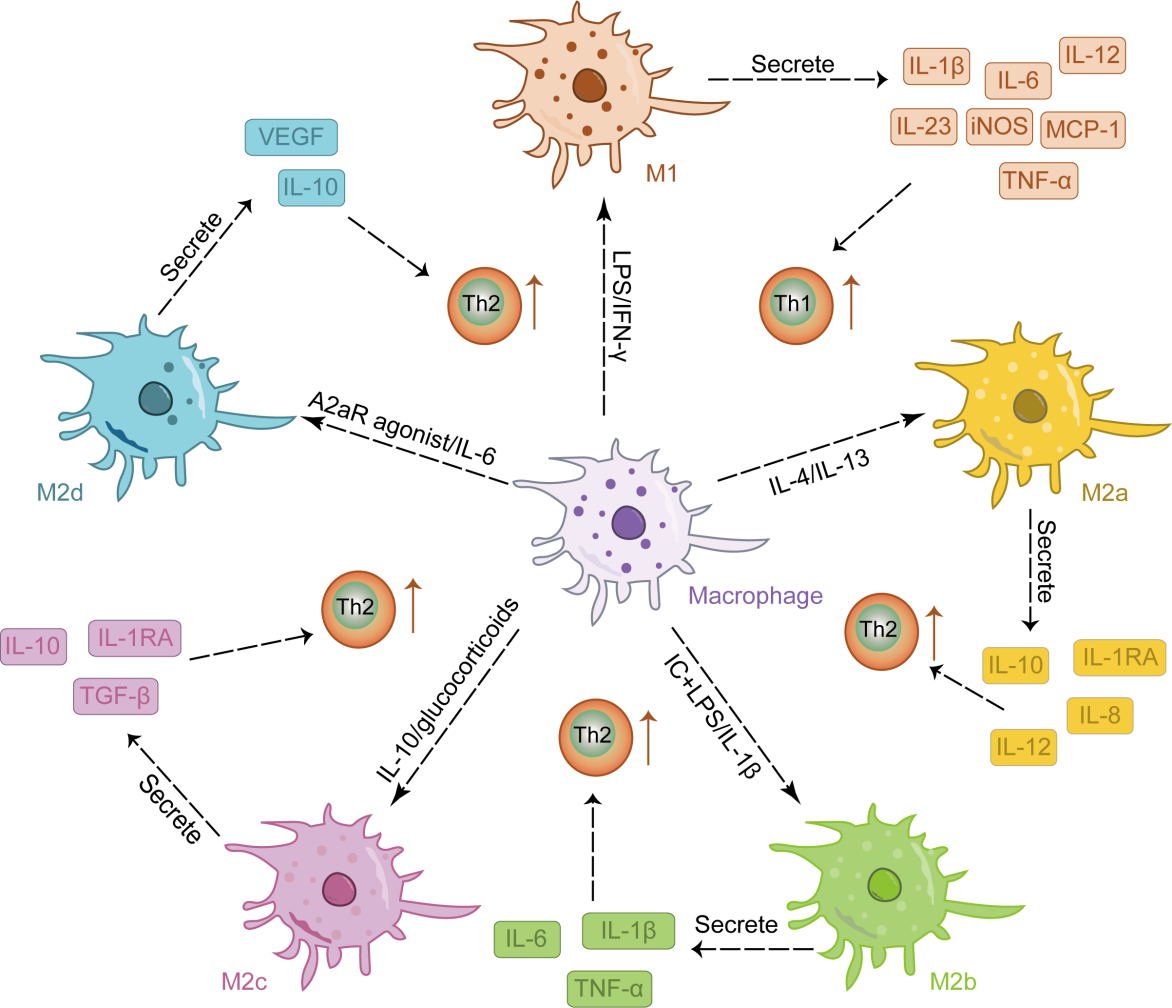
The stimulation of various factors induced the differentiation of macrophages into distinct M1 and M2 phenotypes, each exhibiting a unique cytokine secretion profile to facilitate immune response.

M2 activation occurs in response to stimulation by IL-4, IL-10, and IL-13 (35, 36). As reparative macrophages, M2 macrophages play a crucial role in suppressing inflammatory responses and facilitating tissue repair and wound healing (37, 38). They are characterized by their ability to promote TH2 responses and exhibit overexpression of cytokines such as IL-10, transforming growth factor (TGF-β), vascular endothelial growth factor (VEGF), epidermal growth factor (EGF), and arginase 1(Arg1) (35). M2 macrophages can be further classified into four subtypes: M2a, M2b, M2c, and M2d, each exhibiting distinct functionalities (39). Among these subtypes, IL-4 or IL-13 induces the polarization of M2a macrophages which predominantly express IL-12, interleukin-1 receptor antagonist (IL-1RA), IL-8, and IL-10. This subtype plays a crucial role in anti-inflammatory responses and tissue repair (40–42); Immune complex and LPS/IL-1β stimulation triggers the activation of M2b macrophages leading to increased secretion of pro-inflammatory cytokines such as TNF-α, IL-6, and IL-1β. These cells significantly contribute to immune regulation while also promoting infection and tumor progression (43, 44); Activation of M2c macrophages by either IL-10 or glucocorticoids results in the release of numerous anti-inflammatory cytokines (IL-10, TGF-β, IL-1RA). Primarily involved in tissue remodeling and immunosuppression (45, 46), this subtype is often referred to as “inactivated” macrophages; Lastly, the M2d phenotype arises from synergistic induction by both IL-6 and adenosine A2a receptor agonists; it primarily expresses IL-10 and VEGF, thereby facilitating angiogenesis and tumor growth (42, 47). M2 macrophages are involved in tissue repair and angiogenesis in skin lesions, but M2 hyperpolarization may lead to excessive collagen production and scar formation (48). Therefore, maintaining the balance of M1/M2 macrophages is crucial for restoring the normal skin immune environment, as disrupting this balance can lead to pathological conditions (49, 50). **Figure 1** provides an overview of the phenotype and function of macrophages stimulated by various factors.

As crucial components of the skin’s immune response, macrophages are present in both the dermis and epidermis. Langerhans cells (LCs) in the epidermis develop similarly to macrophages, originating from yolk sac-derived progenitor cells and fetal liver monocytes. They are recruited into the epidermis prenatally, and under homeostatic conditions, LCs undergo self-renewal; only during inflammation do blood monocytes replenish the population of epidermal LCs. LCs serve as the primary barrier of the skin’s immune system, contributing to maintaining skin barrier integrity, immune homeostasis, and limiting viral infections (51–53). Dermal macrophages are also derived from the yolk sac and/or fetal liver. Recent studies have found that CD163 perivascular macrophages and CD64 perinerve macrophages exist in the human dermis. When an injury occurs, dermal macrophages can be rapidly activated to exert antigenic phagocytosis effect (54, 55). Macrophages play a pivotal role in various cutaneous disorders, particularly inflammatory skin conditions (32). Foamy macrophages have been observed abundantly in acne lesions (56), and these cells have been found to express TREM2 (57). Tran et al. utilized single-cell transcriptome analysis to demonstrate the enhanced specificity and abundance of TREM2-expressing macrophages at the site of skin lesions in acne patients (58). Further investigation into the macrophage phenotype in acne revealed a persistent presence of M1-type macrophages within the skin lesions, potentially contributing to chronic inflammation in affected individuals (59).

## 3 Evidence of macrophages involvement in acne

### 3.1 Macrophages and sebum metabolism

Recently, several authors have emphasized the association between acne and metabolic diseases, positing that acne is essentially a metabolic disorder (60, 61). Disruption of sebum metabolism represents the primary characteristic of acne and is also deemed a necessary condition for its onset (62, 63). Sebum consists of triglycerides, free fatty acids, cholesterol, squalene, and other constituents (64). The increase in sebum secretion from related sebaceous glands leads to abnormal keratinization of the follicular sebaceous glands, microbial proliferation, inflammation induction as well as initiation of potential immune mechanisms and inflammatory cascade reactions resulting in skin lesion formation (65).

The squalene content in the skin sebum of acne patients increases to varying degrees (66). Squalene induces macrophages to express TREM2, promoting lipid uptake and catabolic metabolism, thereby enhancing the metabolic activity of macrophages and their phagocytic ability toward lipids and *C. acnes* (56, 67). Knockout of TREM2 in macrophages can inhibit downstream signaling pathways and negatively regulate the recruitment of macrophages, leading to insulin resistance (IR), adipose cell hypertrophy, and lipid accumulation (68). However, it has been observed that squalene epoxide can be generated from squalene by TREM2-expressing macrophages. This conversion results in the formation of a polyunsaturated lipid known as squalene epoxide which possesses ROS-clearing properties. Consequently, this process blocks the antibacterial effect of macrophages directly triggering inflammation and exacerbating acne (69, 70). Concurrently, squalene stimulates TREM2 macrophages to induce the secretion of pro-inflammatory cytokines (IL-18, IL-1β), suppress the production of anti-inflammatory cytokine IL-10, and exacerbate inflammation. These pro-inflammatory factors also contribute to IR and worsen sebum metabolism disorders (15, 71). These findings unveil a distinctive interaction pattern between TREM2 macrophages and sebum metabolism in patients with acne. In summary, disruption of sebum metabolism in acne leads to excessive squalene production, which enhances the phagocytosis of lipids and *C. acnes* by TREM2 macrophages. However, the converted squalene epoxides effectively eliminate ROS, thereby impeding the antimicrobial response of macrophages. Consequently, these TREM2 macrophages fail to reduce bacterial load but instead secrete IL-18 and upregulate chemokine expression, resulting in a disease-specific inflammatory response that further exacerbates sebum metabolism disorder.

Epidemiological evidence suggests a higher prevalence of acne among individuals with obesity compared to those with normal weight, and a positive correlation has been observed between body mass index (BMI) and the severity of acne (72). The presence of excessive peripheral androgens and IR often accompanies obesity, which may contribute to the increased susceptibility of obese patients to acne. In this context, adipose tissue macrophages play a pivotal role in regulating inflammation (73, 74). Studies have demonstrated a significant increase in pro-inflammatory M1 macrophage infiltration in the adipose tissue of obese mice compared to normal-weight mice (75, 76). Obesity can impair the innate immune function of adipose tissue, promoting macrophage polarization toward a pro-inflammatory phenotype and resulting in excessive secretion of TGF-β by mature adipocytes (77), which subsequently induces Th17 cell production (78). Th17 cells are capable of secreting IFN-γ and IL-17, contributing to the formation of acne lesions (79).

The Sterol Regulatory Element-Binding Protein(SREBP), Peroxisome Proliferator-Activated Receptor (PPAR), and Liver X Receptor(LXR) pathways play crucial roles in regulating metabolism and are closely associated with obesity. Increasing evidence has highlighted their involvement in the pathogenesis of acne (80–82). Active LXR can inhibit sebum cell proliferation, stimulate lipid synthesis required for epidermal barrier formation (83), and induce increased sebum production (84). Additionally, LXR activation has been shown to upregulate the expression of inflammatory factors such as IFN-γ and promote M1 macrophage polarization (85).

The SREBP serves as a direct target gene of LXR (86), promoting cholesterol biosynthesis and playing a crucial role in macrophage lipid metabolism (87, 88). In macrophages, SREBP hinders cholesterol efflux through the ATP-binding cassette transporter A1 (87, 89), potentially contributing to foam cell formation in acne. Intriguingly, activation of SREBP induces alterations in cholesterol metabolism that impede affection while inducing pro-inflammatory M1 polarization in macrophages (90). The regulation of sebaceous lipids involves the coordinated action of PPAR and LXR, which play a synergistic role in controlling epidermal growth, differentiation, and lipid metabolism (91, 92). Treatment with PPARγ ligands has been shown to reduce sebum production in human sebaceous glands (93); however, it has also been observed that PPARγ can enhance lipid accumulation in these cells and promote sebum secretion when patients are treated with PPARγ agonists (91, 94). Additionally, activation of PPARγ inhibits immune activation markers such as TNF-α, IL-6, and IL-1β in mouse macrophages (95).

**Figure 2:**
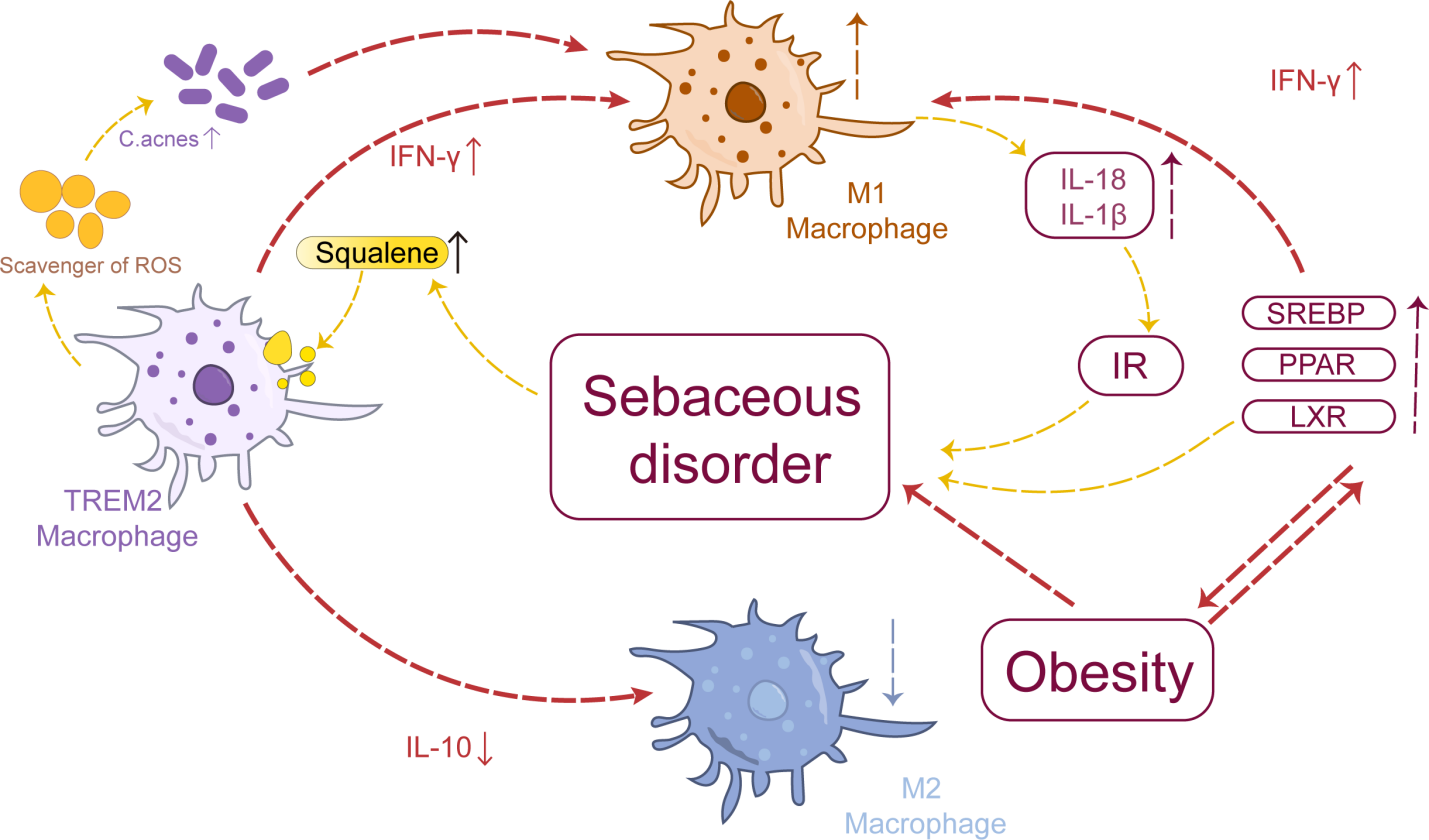
Macrophages and sebum metabolism: Aberrant sebum metabolism promotes the polarization of TREM2 macrophages toward the M1 phenotype, thereby inducing the upregulation of pro-inflammatory factors such as IL-18 and IL-1β. These cytokines themselves can contribute to IR, exacerbating cortical metabolic disorders. Sebum metabolism is intricately linked to obesity, which can drive the polarization of adipose tissue macrophages toward the M1 phenotype, influencing SREBP, PPAR, and LXR pathways and ultimately impacting sebum metabolism.

Acne patients frequently experience complications such as IR, obesity, and disturbances in glucose and lipid metabolism, which collectively contribute to heightened levels of M1 macrophages in sebum, thereby exacerbating tissue inflammation and metabolic disorders that further promote acne development (**Figure 2**).

### 3.2 Macrophages and abnormal androgen regulation

Abnormal regulation of androgens plays a pivotal role in the pathogenesis of acne. Women with acne generally exhibit higher levels of serum testosterone and dihydrotestosterone compared to non-acne patients, albeit within the normal range (96, 97). A questionnaire survey involving 400 women revealed that over 50% of acne patients reported experiencing premenstrual episodes (98), suggesting that fluctuations in androgen production rather than solely excessive androgen production are likely to be the triggering factor for acne. The sebaceous gland serves as a pivotal target organ for androgens. Sebum secretion and the development of sebaceous glands are directly regulated by androgens, particularly testosterone (T), which directly controls sebaceous glands. T is enzymatically converted into dihydrotestosterone (DHT) by type I 5α-reductase (99). DHT promotes the proliferation of sebaceous cells, augments sebum secretion, and induces the release of pro-inflammatory factors. However, when there is an improper excretion of increased sebum production, it accumulates within hair follicles forming lipid thrombi that combine with *C. acnes* invasion to trigger acne formation (100–102). The regulation of androgens on sebaceous glands is closely related to the affinity between androgens and androgen receptors (AR). Moreover, AR can independently regulate the development of sebaceous glands, stimulate excessive secretion, up-regulate certain inflammatory factors, promote inflammatory response, and contribute to acne pathogenesis (103, 104).

The binding of active androgens to AR can stimulate macrophages to secrete pro-inflammatory factors IL-1, IL-6, and TNF-α (105), which is consistent with the inflammatory response observed at acne lesion sites. Androgen precursors can inhibit the secretion of corticotropin-releasing hormone and adrenocorticotropin through feedback regulation, reducing the level of α-melanocyte stimulating hormone that possesses anti-inflammatory effects, thereby indirectly promoting the release of pro-inflammatory factors (102). It has been demonstrated that IL-6 and TNF-α can stimulate androgen synthesis and secretion (106). Therefore, it can be inferred that dysregulated androgen regulation leads to macrophage-mediated secretion of pro-inflammatory factors in acne lesions, inducing M1 macrophage polarization, local inflammatory responses, further exacerbating abnormal androgen levels, forming a vicious cycle contributing to the onset and progression of acne symptoms.

Recent studies have revealed a close association between acne and IR as well as hyperinsulinemia, with the severity of acne exhibiting a consistent correlation (107). Hyperinsulinemia stimulates the IGF-1 receptor, leading to increased expression of IGF-1, augments adrenal sensitivity to adrenocorticotropic hormone, facilitates adrenal androgen production, enhances AR activity, and subsequently manifests clinical characteristics indicative of aberrant androgen regulation (108). The occurrence of IR is intricately linked to inflammation (73). Macrophages, as the primary source of inflammatory mediators, can secrete pro-inflammatory factors such as TNF-α to facilitate IR formation by augmenting serine phosphorylation of insulin receptor substrate-1 (109). Under conditions characterized by IR or inflammation, macrophage-mediated insulin signaling phosphorylates forkhead box O1(FOXO1) and promotes nuclear exclusion. The heightened activity of FOXO1 further stimulates excessive IL-1β production in macrophages (110), which potentially exacerbates IR and contributes to abnormal androgen regulation. Inhibiting FOXO1 in macrophages can induce polarization toward an M2-like phenotype in lipopolysaccharide-stimulated macrophages, thereby reducing inflammatory response, alleviating IR, and diminishing androgen secretion along with AR activity (111, 112). In acne pathogenesis, IR may promote the upregulation of FOXO1 expression in macrophages leading to their polarization toward an M1 phenotype instead of an M2 phenotype. This phenomenon subsequently aggravates IR and disrupts normal androgen regulation.

In summary, dysregulated androgen signaling promotes the polarization of cutaneous macrophages toward the M1 phenotype, subsequently inducing the upregulation of pro-inflammatory cytokines such as IL-6 and TNF-α, thereby triggering a local inflammatory response. This cascade further enhances androgen synthesis and secretion, ultimately leading to acne development.

### 3.3 Macrophages and *C. acnes*

The bacterium *C. acnes* is widely distributed on the skin and constitutes the predominant microbe in the sebaceous glands of hair follicles, accounting for approximately 89% (113). *C. acnes* has the ability to produce endogenous porphyrins that oxidize squalene, thereby promoting acne vulgaris formation in keratinocytes (114). On the other hand, *C. acnes* can hydrolyze sebum into glycerol and free fatty acids, stimulating hair follicles and exacerbating acne (115). Mice exposed to an excess of *C. acnes* exhibit symptoms resembling human acne, including redness and erythema. Microscopic examination reveals a significant increase in infiltrating inflammatory cells such as macrophages (116). Consequently, exposure to excessive amounts of *C. acnes* is commonly employed to establish animal models mimicking acne-like conditions (117).

According to extensive *in vivo* and *in vitro* investigations, it has been observed that *C. acnes* infiltration into sebaceous glands leads to the secretion of a diverse range of bioactive enzymes, including lipase. This enzyme is capable of hydrolyzing triglycerides present in sebum, resulting in the generation of free fatty acids and low molecular weight polypeptides. The activation of toll-like receptors (TLRs) on keratinocytes and sebum cells by these low molecular weight peptides subsequently upregulates the expression levels of TLR2 and TLR4. Additionally, this process triggers macrophages to adopt an M1 phenotype and release pro-inflammatory cytokines (such as IL-1 and TNF-α), which play pivotal roles in acne-associated inflammatory responses (118–120). IL-1β plays a pivotal role in the initiation of acne inflammation (121). Simultaneously, pro-inflammatory cytokines like IL-1β and TNF-α can also induce excessive production of proteolytic enzymes (e.g., matrix metalloproteinases), leading to collagen fiber degradation, impaired recombination, and subsequent alterations in local skin tissue structure during the reparative phase of post-acne erythema, ultimately resulting in scar formation (122, 123).

In summary, *C. acnes* in the skin can induce macrophages to polarize into M1 type and secrete pro-inflammatory factors, thereby driving the inflammatory response associated with acne. On the other hand, these pro-inflammatory factors contribute to collagen fiber degradation and recombination, ultimately leading to the development of acne erythema.

## 4 Natural plant products modulate the influence of macrophages in acne

### 4.1 Quercetin

Quercetin is a natural flavonoid of significant medicinal value, characterized by its yellow needle-like crystal monomer (124). It is widely distributed in Chinese herbal medicine as well as various vegetables and fruits. This compound exhibits diverse biological and pharmacological effects, encompassing anti-inflammatory, antioxidant, antibacterial, and cardiovascular protective properties (125). The anti-inflammatory and antibacterial effects of quercetin have shown promising potential in the treatment of acne (126, 127). Studies have demonstrated that quercetin effectively inhibits the upregulation of pro-inflammatory cytokines including IL-1β, IL-8, IL-6, and TNF-α in *C. acnes*-stimulated macrophages. Furthermore, quercetin significantly mitigated skin erythema induced by *C. acnes*. The study also revealed that quercetin exerts an inhibitory effect on TLR-2, a key innate immune receptor, thereby influencing the functioning of the innate immune system. Although the specific phenotype of macrophages stimulated by quercetin was not specified in this study, considering TLR-2’s high expression on macrophage surfaces and its impact on macrophage polarization, it is plausible to suggest that quercetin promotes the polarization of macrophages toward the M2 phenotype while reducing their M1 phenotype. Consequently, we propose that quercetin can effectively suppress pro-inflammatory factor secretion by M1 macrophages in acne (128).

### 4.2 Baicalin

Baicalin is a lipophilic flavonoid glycoside isolated from Scutellaria baicalensis (129). It has anti-inflammatory, antioxidant, cardioprotective, anticancer, and antiviral effects (130, 131). It was found that baicalin significantly decreased the expression of M1 macrophage-related cytokines such as IL-1β, IL-6, TNF-α and NLRP3 in acne models, and inhibited the M1 polarization of macrophages by inhibiting the nuclear translocation of NF-κB p65. Consequently, it is anticipated to emerge as a viable therapeutic option for the management of acne (132).

### 4.3 Schisandrin

Schisandrin (SCH) is a lignan-like compound that is the main bioactive component isolated from Schisandra chinensis (133). It has been reported to have a variety of biological activities, such as anti-inflammatory, hepatoprotective, anti-tumor, and antioxidant, and affects the respiratory, cardiovascular, and central nervous systems (134). In addition, SCH has been found to prevent lipopolysaccharide-induced inflammation (135). Therefore, some scholars have studied the mechanism of SCH to improve acne. Firstly, it was observed that SCH significantly suppressed caspase-1 activation and downregulated the expression of the inflammatory cytokine IL-1β. Moreover, SCH exhibited the ability to attenuate NLRP3 levels by inhibiting mitochondrial ROS production, ATP release, and K efflux, thereby impeding NLRP3 inflammasome activation. In conclusion, we propose that SCH exerts its acne-inhibitory effects by restraining the phenotypic transformation of M1 macrophages and mitigating inflammatory infiltration (136).

Acne comorbidities suggest that diabetes mellitus is closely related to acne, and it has been found that SCH C can attenuate diabetic nephropathy by regulating macrophage M1 to M2 polarisation via Swiprosin-1/IFN-γ-Rβ, which could also potentially serve as a future target for SCH in the treatment of acne (137).

### 4.4 Licochalcone A

The compound licochalcone A (LicA), derived from glycyrrhizin (138), is a flavonoid with diverse pharmacological effects including anti-inflammatory, antimicrobial, and antioxidant properties in the skin (139). Cai et al. (140) found that LicA significantly inhibited the binding of LPS and TLR4 and blocked the activation of signaling pathways downstream of TLR4, including MAPK and NF-κB, thereby suppressing macrophage inflammation. Ludger et al. (141) found similar anti-inflammatory effects of LicA in a model of skin irritation, and the researchers further demonstrated that moisturizers containing LicA can ameliorate disease severity in patients with mild to moderate acne (142). Subsequent investigations have further elucidated the mechanism of action of LicA on acne, revealing its potential for alleviating ear inflammation and swelling symptoms in mice with acne models. *In vitro*, experiments utilizing bone marrow-derived macrophage (BMDM) cells have indicated that LicA hampers NLRP3 inflammasome activation in *C. acnes*-induced macrophages by impeding mitochondrial ROS production. LicA inhibits the production of ROS in macrophages, which are secreted by M1-type macrophages and can further drive M1 polarisation, so we hypothesized that LicA could inhibit M1-type macrophage polarisation by inhibiting ROS production (143).

### 4.5 Phenolic compounds from Quercus acutissima Carruth. leaves

The perennial tree Quercus acutissima Carruth., belonging to the Fagaceae family, exhibits anti-allergic, anti-inflammatory, and anti-edema properties, making it an effective treatment for conditions such as eczema, boils, diarrhea, and tonsillitis (144). The researchers conducted a comparative analysis of the effects of phenolic compounds derived from Quercus acutissima Carruth. leaves, namely hyperoside, astragalin, kaempferol 3-O-(6”-galloyl)-β-D-glucopyranoside, quercetin 3-O-(6”-O-galloyl)-β-D-glucopyranoside, pedunculagin, and casuarinin on acne. Among these compounds, casuarinin exhibited superior efficacy. *In vitro* assays using RAW264.7 cells demonstrated that the extracts effectively suppressed the production of NLRP3, IL-1β, and 5α reductase in macrophages. This effect can be attributed to the inhibitory action of phenolic compounds present in Quercus acutissima Carruth. leaves on M1 macrophage polarization and subsequent reduction in inflammatory response (145).

### 4.6 Cembrene diterpenoids from the cultured soft coral sinularia flexibilis

Diterpenoids, the most abundant class of metabolites in soft corals, exhibit diverse biological activities including anti-tumor, anti-inflammatory, and antibacterial effects (146). The diterpenoid compound derived from Taiwan soft coral has been discovered to possess inhibitory effects on the expression of pro-inflammatory proteins, including iNOS and cyclohexane-2 (COX-2), in LPS-stimulated macrophages (147). Six cembrene diterpenoids were isolated from cultured soft coral sinularia flexibilis by researchers, who observed their significant reduction of ear edema induced by *C. acnes* in rats. Moreover, these compounds demonstrated a decrease in the expression of inducible NO synthase in RAW264.7 cells. We propose that cembrene diterpenoids inhibit M1 macrophage polarization, thereby reducing NO production during this process. Additionally, cembrene diterpenoids were found to significantly inhibit the phosphorylation of related proteins within the MAPK pathway in skin lesions. Therefore, the therapeutic potential of cultured soft coral Sinularia flexibilis-derived cembrene diterpenoids for acne treatment is supported and they play an active role as an alternative therapy for acne patients (148).

### 4.7 Meconopsis quintuplinervia Regel

The Meconopsis quintuplinervia Regel (MQ), a plant belonging to the genus Meconopsis Vig in the Papaveraceae family, predominantly thrives in the Tibet Autonomous Region. Initial investigations have revealed that its active extract primarily comprises flavonoids and polyphenols. According to Tibetan Medicine Annals, it is documented that MQ possesses properties such as heat-clearing and detoxification, antioxidation, anti-inflammatory, and analgesic effects. It is commonly employed for treating ailments including headaches, hepatitis, pneumonia, and edema, among others (149). In a murine model of acne, it has been observed that MQ mitigates erythema and inflammatory infiltration in ear acne. Furthermore, the expression of M1-related cytokines (IL-6, IL-1β, and TNF-α) was reduced in an inflammatory macrophage cell model, while phosphorylation of proteins associated with the MAPKs pathway such as ERK and JNK was significantly inhibited in RAW264.7 cells. Additionally, nuclear translocation of NF-κB p65 was suppressed. Hence, MQ exhibits therapeutic potential by modulating macrophage infiltration and polarization within acne lesions (150).

### 4.8 Kaempferia parviflora extracts

The herbaceous plant Kaempferia parviflora (KP), a member of the ginger family (151), exhibits anti-inflammatory, anti-allergic, and anti-obesity effects (152). It is rich in various flavonoids such as 5,7-dimethoxy-flavonoids, 5-hydroxy-3,7,4’- trimethoxyflavone, and 5-hydroxy-3,7- dimethoxyflavone (153), which have been implicated in metabolic diseases, cognitive disorders, and cancer research (154). The extracts of Kaempferia parviflora (KP) were observed to attenuate the expression of inflammatory cytokines, specifically TNF-α, in lipopolysaccharide-stimulated macrophages. Additionally, it exhibited inhibitory effects on NO production and downregulated the expression of iNOS and COX-2. Furthermore, it suppressed the phosphorylation of IĸBα and NF-κB as well as the enhanced nuclear translocation of NF-κB p65. Lastly, KP extracts demonstrated an inhibitory effect on sebum cell-mediated lipid synthesis. In conclusion, KP extracts possess potential anti-acne properties by modulating M1 macrophage polarization and reducing inflammatory infiltration (155).

### 4.9 Jumihaidokuto

Jumihaidokuto (JHT) is a traditional Japanese-Chinese herbal medicine formulated with platycodon root, dandelion, bamboo leaves, bupleurum root, snake seed root, tuckahoe, oak bark, licorice, Schisandra spicule and ginger (156). The herb possesses heat-clearing and detoxifying properties, commonly employed in the treatment of suppurative dermatosis, urticaria, eczema, and athlete’s foot among other ailments (156, 157). JHT has demonstrated efficacy in inhibiting acne breakouts (157). Experimental findings indicate that JHT significantly enhances the expression of M2-associated CD206 cells as well as M1-associated CD86 and CD192 markers, suggesting a non-selective activation of macrophages rather than targeting specific subtypes. Considering its observed inhibition of rat ear acne model rashes at 24 hours and beyond, it is hypothesized that macrophage activation may contribute to the reduction or acceleration of dermatitis regression; however, further investigation is required to elucidate the underlying mechanism (158).

## 5 Conclusion

The pathological features of acne include inflammation, disturbance of sebum metabolism, abnormal androgen regulation, colonization of *C. acnes*, and abnormal keratosis of hair follicles and sebaceous glands (159–163). These characteristics are not independent but rather intertwined and mutually causal (164). With the continuous advancement of research on macrophages in various fields, including inflammation and metabolism, our understanding of macrophages is expanding. As summarized in this paper, the close association between macrophages and acne suggests a reciprocal relationship between these two fields. In the context of acne pathogenesis, macrophages polarize into pro-inflammatory M1 type and secrete pro-inflammatory cytokines, thereby connecting these underlying mechanisms. Although there have been sufficient studies on the role of macrophages in sebum metabolism disorder, abnormal androgen regulation, and colonization of *C. acnes* as independent pathogenic factors (165, 166), their specific involvement in acne remains limited, and further studies are needed to understand the specific mechanisms of macrophage regulation in acne. In recent years, the occurrence and mechanisms of macrophages in the context of inflammatory skin disease have been a focal point of research in the field of dermatology. Further investigation into the mechanism of macrophages in acne will not only advance our knowledge of acne but also shed light on the prevention and treatment of other inflammatory skin disease, such as rosacea (167, 168).

Studies conducted in the past have indicated that natural plant products possess therapeutic properties that can be beneficial in treating acne. However, fewer studies have been conducted on the therapeutic effects of natural plant products on acne through macrophage modulation, focusing mainly on flavonoids, phenols, lignans, and other compound classes. Most of these studies indirectly indicate that they can treat acne through macrophage targeting by investigating the inhibition of M1 macrophage-associated cytokine expression by natural compounds. Moreover, most of these studies are based on animal experiments and lack validation in large-scale, long-term clinical studies. These limitations highlight the need for further research to bridge the gap between basic experiments and clinical translation. We encourage researchers to further explore and elucidate the signaling pathways associated with macrophages, to study the mechanisms linking macrophages to the pathological process of acne, and to further investigate the key targets of natural plant compounds that regulate macrophages, so as to provide value for the development of new drugs and to further provide new ideas and effective strategies for the treatment of acne and even inflammatory skin diseases in the future.

## Data availability statement

The original contributions presented in the study are included in the article/supplementary material. Further inquiries can be directed to the corresponding author.

## Author contributions

DZ: Data curation, Conceptualization, Investigation, Writing – original draft. YW: Data curation, Investigation, Writing – review & editing. SW: Data curation, Writing – review & editing. XJ: Data curation, Writing – review & editing. KG: Data curation, Writing – review & editing. HZ: Data curation, Writing – review & editing. MZ: Funding acquisition, Writing – review & editing.

## References

[1] Dréno B Dagnelie MA Khammari A Corvec S. The skin microbiome: A new actor in inflammatory acne. Am J Clin Dermatol. (2020) 21:18–24. doi: 10.1007/s40257-020-00531-1 32910436 PMC7584556

[2] Sun L Wang Q Wang H Huang J Yu Z. A cross-sectional cohort study on the skin microbiota in patients with different acne durations. Exp Dermatol. (2023) 32:2102–11. doi: 10.1111/exd.14951 37846925

[3] Mias C Thouvenin MD Gravier E Dalmon S Bouyer K Alvare S. Change in *Cutibacterium acnes* phylotype abundance and improvement of clinical parameters using a new dermo-cosmetic product containing Myrtus communis and Celastrol enriched plant cell culture extracts in patients with acne vulgaris. J Eur Acad Dermatol Venereol. (2023) 37:20–5. doi: 10.1111/jdv.18792 36729402

[4] Kostecka M Kostecka J Szwed-Gułaga O Jackowska I Kostecka-Jarecka J. The impact of common acne on the well-being of young people aged 15-35 years and the influence of nutrition knowledge and diet on acne development. Nutrients. (2022) 14:5293. doi: 10.3390/nu14245293 36558452 PMC9784447

[5] Gieler U Gieler T Kupfer JP. Acne and quality of life - impact and management. J Eur Acad Dermatol Venereol. (2015) 29:12–4. doi: 10.1111/jdv.13191 26059729

[6] Fife D. Evaluation of acne scars: how to assess them and what to tell the patient. Dermatol Clinics. (2016) 34:207–13. doi: 10.1016/j.det.2015.11.009 27015781

[7] Halvorsen JA Dalgard F Thoresen M Bjertness E Lien L. Is the association between acne and mental distress influenced by diet? Results from a cross-sectional population study among 3775 late adolescents in Oslo, Norway. BMC Public Health. (2009) 9:340. doi: 10.1186/1471-2458-9-340 19758425 PMC2751780

[8] Oge LK Broussard A Marshall MD. Acne vulgaris: diagnosis and treatment. Am Family Physician. (2019) 100:475–84. 31613567

[9] Leyden J Stein-Gold L Weiss J. Why topical retinoids are mainstay of therapy for acne. Dermatol Ther. (2017) 7:293–304. doi: 10.1007/s13555-017-0185-2 PMC5574737 28585191

[10] Chlebus E Serafin M Chlebus M. Is maintenance treatment in adult acne important? Benefits from maintenance therapy with adapalene, and low doses of alpha and beta hydroxy acids. J Dermatol Treat. (2019) 30:568–71. doi: 10.1080/09546634.2018.1484874 29873567

[11] Dreno B Dekio I Baldwin H Demessant AL Dagnelie MA Khammari A. Acne microbiome: From phyla to phylotypes. J Eur Acad Dermatol Venereol. (2023) 00:1–8. doi: 10.1111/jdv.19540 37777343

[12] Melnik BC. Acne transcriptomics: fundamentals of acne pathogenesis and isotretinoin treatment. Cells. (2023) 12:2600. doi: 10.20944/preprints202310.0472.v1 37998335 PMC10670572

[13] Zouboulis CC Jourdan E Picardo M. Acne is an inflammatory disease and alterations of sebum composition initiate acne lesions. J Eur Acad Dermatol Venereol. (2014) 28:527–32. doi: 10.1111/jdv.12298 24134468

[14] Byrd AL Belkaid Y Segre JA. The human skin microbiome. Nat Rev Microbiol. (2018) 16:143–55. doi: 10.1038/nrmicro.2017.157 29332945

[15] Lovászi M Mattii M Eyerich K Gácsi A Csányi E Kovács D. Sebum lipids influence macrophage polarization and activation. Br J Dermatol. (2017) 177:1671–82. doi: 10.1111/bjd.15754 28646583

[16] Suvanprakorn P Tongyen T Prakhongcheep O Laoratthaphong P Chanvorachote P. Establishment of an anti-acne vulgaris evaluation method based on TLR2 and TLR4-mediated interleukin-8 production. In Vivo. (2019) 33:1929–34. doi: 10.21873/invivo.11687 PMC6899138 31662521

[17] van Furth R Sluiter W. Distribution of blood monocytes between a marginating and a circulating pool. J Exp Med. (1986) 163:474–9. doi: 10.1084/jem.163.2.474 PMC2188035 3944542

[18] Mosser DM Edwards JP. Exploring the full spectrum of macrophage activation. Nat Rev Immunol. (2008) 8:958–69. doi: 10.1038/nri2448 PMC2724991 19029990

[19] Zhang Z Huang L Brayboy L. Macrophages: an indispensable piece of ovarian health. Biol Reproduction. (2021) 104:527–38. doi: 10.1093/biolre/ioaa219 PMC7962765 33274732

[20] Ruytinx P Proost P Van Damme J Struyf S. Chemokine-induced macrophage polarization in inflammatory conditions. Front Immunol. (2018) 9:1930. doi: 10.3389/fimmu.2018.01930 30245686 PMC6137099

[21] Lee CZW Ginhoux F. Biology of resident tissue macrophages. Development. (2022) 149:dev200270. doi: 10.1242/dev.200270 35502781

[22] Ginhoux F Guilliams M Naik SH. Editorial: dendritic cell and macrophage nomenclature and classification. Front Immunol. (2016) 7:168. doi: 10.3389/fimmu.2016.00168 27199991 PMC4852170

[23] Chen X Liu Y Gao Y Shou S Chai Y. The roles of macrophage polarization in the host immune response to sepsis. Int Immunopharmacol. (2021) 96:107791. doi: 10.1016/j.intimp.2021.107791 34162154

[24] Nathan CF Murray HW Wiebe ME Rubin BY. Identification of interferon-gamma as the lymphokine that activates human macrophage oxidative metabolism and antimicrobial activity. J Exp Med. (1983) 158:670–89. doi: 10.1084/jem.158.3.670 PMC2187114 6411853

[25] Mills CD Kincaid K Alt JM Heilman MJ Hill AM. M-1/M-2 macrophages and the Th1/Th2 paradigm. J Immunol. (2000) 164:6166–73. doi: 10.4049/jimmunol.164.12.6166 10843666

[26] Viola A Munari F Sánchez-Rodríguez R Scolaro T Castegna A. The metabolic signature of macrophage responses. Front Immunol. (2019) 10:1462. doi: 10.3389/fimmu.2019.01462 31333642 PMC6618143

[27] Murray PJ Allen JE Biswas SK Fisher EA Gilroy DW Goerdt S. Macrophage activation and polarization: nomenclature and experimental guidelines. Immunity. (2014) 41:14–20. doi: 10.1016/j.immuni.2014.06.008 25035950 PMC4123412

[28] Ginhoux F Schultze JL Murray PJ Ochando J Biswas SK. New insights into the multidimensional concept of macrophage ontogeny, activation, and function. Nat Immunol. (2016) 17:34–40. doi: 10.1038/ni.3324 26681460

[29] West AP Brodsky IE Rahner C Woo DK Erdjument-Bromage H Tempst P. TLR signalling augments macrophage bactericidal activity through mitochondrial ROS. Nature. (2011) 472:476–80. doi: 10.1038/nature09973 PMC3460538 21525932

[30] Bashir S Sharma Y Elahi A Khan F. Macrophage polarization: the link between inflammation and related diseases. Inflammation Res. (2016) 65:1–11. doi: 10.1007/s00011-015-0874-1 26467935

[31] Mommert S Hüer M Schaper-Gerhardt K Gutzmer R Werfel T. Histamine up-regulates oncostatin M expression in human M1 macrophages. Br J Pharmacol. (2020) 177:600–13. doi: 10.1111/bph.14796 PMC7012943 31328788

[32] Sun Q Hu S Lou Z Gao J. The macrophage polarization in inflammatory dermatosis and its potential drug candidates. Biomed Pharmacother. (2023) 161:114469. doi: 10.1016/j.biopha.2023.114469 37002572

[33] Louiselle AE Niemiec SM Zgheib C Liechty KW. Macrophage polarization and diabetic wound healing. Trans Res: J Lab Clin Med. (2021) 236:109–16. doi: 10.1016/j.trsl.2021.05.006 34089902

[34] Das LM Binko AM Traylor ZP Peng H Lu KQ. Vitamin D improves sunburns by increasing autophagy in M2 macrophages. Autophagy. (2019) 15:813–26. doi: 10.1080/15548627.2019.1569298 PMC6526871 30661440

[35] Wang LX Zhang SX Wu HJ Rong XL Guo J. M2b macrophage polarization and its roles in diseases. J Leukocyte Biol. (2019) 106:345–58. doi: 10.1002/JLB.3RU1018-378RR PMC7379745 30576000

[36] Colin S Chinetti-Gbaguidi G Staels B. Macrophage phenotypes in atherosclerosis. Immunol Rev. (2014) 262:153–66. doi: 10.1111/imr.12218 25319333

[37] Yadav S Dwivedi A Tripathi A. Biology of macrophage fate decision: Implication in inflammatory disorders. Cell Biol Int. (2022) 46:1539–56. doi: 10.1002/cbin.11854 35842768

[38] Xia T Fu S Yang R Yang K Lei W Yang Y. Advances in the study of macrophage polarization in inflammatory immune skin diseases. J Inflammation. (2023) 20:33. doi: 10.1186/s12950-023-00360-z PMC10568804 37828492

[39] Anders CB Lawton TMW Ammons MCB. Metabolic immunomodulation of macrophage functional plasticity in nonhealing wounds. Curr Opin Infect Dis. (2019) 32:204–9. doi: 10.1097/QCO.0000000000000550 PMC7024605 30950855

[40] Kolosowska N Keuters MH Wojciechowski S Keksa-Goldsteine V Laine M Malm T. Peripheral administration of IL-13 induces anti-inflammatory microglial/macrophage responses and provides neuroprotection in ischemic stroke. Neurotherapeutics. (2019) 16:1304–19. doi: 10.1007/s13311-019-00761-0 PMC6985054 31372938

[41] Lescoat A Lelong M Jeljeli M Piquet-Pellorce C Morzadec C Ballerie A. Combined anti-fibrotic and anti-inflammatory properties of JAK-inhibitors on macrophages *in vitro* and *in vivo*: Perspectives for scleroderma-associated interstitial lung disease. Biochem Pharmacol. (2020) 178:114103. doi: 10.1016/j.bcp.2020.114103 32562787

[42] Kong X Gao J. Macrophage polarization: a key event in the secondary phase of acute spinal cord injury. J Cell Mol Med. (2017) 21:941–54. doi: 10.1111/jcmm.13034 PMC5387136 27957787

[43] Byrne AJ Mathie SA Gregory LG Lloyd CM. Pulmonary macrophages: key players in the innate defence of the airways. Thorax. (2015) 70:1189–96. doi: 10.1136/thoraxjnl-2015-207020 26286722

[44] Bianchini R Roth-Walter F Ohradanova-Repic A Flicker S Hufnagl K Fischer MB. IgG4 drives M2a macrophages to a regulatory M2b-like phenotype: potential implication in immune tolerance. Allergy. (2019) 74:483–94. doi: 10.1111/all.13635 PMC6492166 30338531

[45] Ohlsson SM Linge CP Gullstrand B Lood C Johansson A Ohlsson S. Serum from patients with systemic vasculitis induces alternatively activated macrophage M2c polarization. Clin Immunol. (2014) 152:10–9. doi: 10.1016/j.clim.2014.02.016 24631966

[46] Horckmans M Ring L Duchene J Santovito D Schloss MJ Drechsler M. Neutrophils orchestrate post-myocardial infarction healing by polarizing macrophages towards a reparative phenotype. Eur Heart J. (2017) 38:187–97. doi: 10.1093/eurheartj/ehw002 28158426

[47] Cao W Peters JH Nieman D Sharma M Watson T Yu J. Macrophage subtype predicts lymph node metastasis in oesophageal adenocarcinoma and promotes cancer cell invasion *in vitro* . Br J Cancer. (2015) 113:738–46. doi: 10.1038/bjc.2015.292 PMC4559839 26263481

[48] Chen L Wang J Li S Yu Z Liu B Song B. The clinical dynamic changes of macrophage phenotype and function in different stages of human wound healing and hypertrophic scar formation. Int Wound J. (2019) 16:360–9. doi: 10.1111/iwj.13041 PMC7948805 30440110

[49] Gosselin D Link VM Romanoski CE Fonseca GJ Eichenfield DZ Spann NJ. Environment drives selection and function of enhancers controlling tissue-specific macrophage identities. Cell. (2014) 159:1327–40. doi: 10.1016/j.cell.2014.11.023 PMC4364385 25480297

[50] Lavin Y Winter D Blecher-Gonen R David E Keren-Shaul H Merad M. Tissue-resident macrophage enhancer landscapes are shaped by the local microenvironment. Cell. (2014) 159:1312–26. doi: 10.1016/j.cell.2014.11.018 PMC4437213 25480296

[51] West HC Bennett CL. Redefining the role of langerhans cells as immune regulators within the skin. Front Immunol. (2017) 8:1941. doi: 10.3389/fimmu.2017.01941 29379502 PMC5770803

[52] Mass E Ballesteros I Farlik M Halbritter F Günther P Crozet L. Specification of tissue-resident macrophages during organogenesis. Science. (2016) 353:aaf4238. doi: 10.1126/science.aaf4238 27492475 PMC5066309

[53] Sim SL Kumari S Kaur S Khosrotehrani K. Macrophages in skin wounds: functions and therapeutic potential. Biomolecules. (2022) 12:1659. doi: 10.3390/biom12111659 36359009 PMC9687369

[54] Kolter J Feuerstein R Zeis P Hagemeyer N Paterson N d'Errico P. A subset of skin macrophages contributes to the surveillance and regeneration of local nerves. Immunity. (2019) 50:1482–97.e7. doi: 10.1016/j.immuni.2019.05.009 31201094

[55] Barreiro O Cibrian D Clemente C Alvarez D Moreno V Valiente Í. Pivotal role for skin transendothelial radio-resistant anti-inflammatory macrophages in tissue repair. Elife. (2016) 5:e15251. doi: 10.7554/eLife.15251 27304075 PMC4961461

[56] Jiang H Li C. Common pathogenesis of acne vulgaris and atherosclerosis. Inflammation. (2019) 42:1–5. doi: 10.1007/s10753-018-0863-y 30073565

[57] Siegel-Axel D Daub K Seizer P Lindemann S Gawaz M. Platelet lipoprotein interplay: trigger of foam cell formation and driver of atherosclerosis. Cardiovasc Res. (2008) 78:8–17. doi: 10.1093/cvr/cvn015 18218686

[58] Do TH Ma F Andrade PR Teles R de Andrade Silva BJ Hu C. TREM2 macrophages induced by human lipids drive inflammation in acne lesions. Sci Immunol. (2022) 7:eabo2787. doi: 10.1126/sciimmunol.abo2787 35867799 PMC9400695

[59] Chiossone L Conte R Spaggiari GM Serra M Romei C Bellora F. Mesenchymal stromal cells induce peculiar alternatively activated macrophages capable of dampening both innate and adaptive immune responses. Stem Cells. (2016) 34:1909–21. doi: 10.1002/stem.2369 27015881

[60] Wang Y Zhu M Wu S Zheng H. Acne comorbidities. Clin Cosmetic Investigational Dermatol. (2022) 15:2415–20. doi: 10.2147/CCID.S392165 PMC9661895 36387963

[61] Biagi LG Sañudo A Bagatin E. Severe acne and metabolic syndrome: A possible correlation. Dermatology. (2019) 235:456–62. doi: 10.1159/000501986 31484190

[62] Melnik BC. Acne vulgaris: The metabolic syndrome of the pilosebaceous follicle. Clinics Dermatol. (2018) 36:29–40. doi: 10.1016/j.clindermatol.2017.09.006 29241749

[63] Clayton RW Göbel K Niessen CM Paus R van Steensel MAM Lim X. Homeostasis of the sebaceous gland and mechanisms of acne pathogenesis. Br J Dermatol. (2019) 181:677–90. doi: 10.1111/bjd.17981 31056753

[64] Mijaljica D Townley JP Spada F Harrison IP. The heterogeneity and complexity of skin surface lipids in human skin health and disease. Prog Lipid Res. (2023) 93:101264. doi: 10.1016/j.plipres.2023.101264 37940006

[65] Kurokawa I Danby FW Ju Q Wang X Xiang LF Xia L. New developments in our understanding of acne pathogenesis and treatment. Exp Dermatol. (2009) 18:821–32. doi: 10.1111/j.1600-0625.2009.00890.x 19555434

[66] Pappas A Johnsen S Liu JC Eisinger M. Sebum analysis of individuals with and without acne. Dermato-endocrinology. (2009) 1:157–61. doi: 10.4161/derm.1.3.8473 PMC2835908 20436883

[67] Nugent AA Lin K van Lengerich B Lianoglou S Przybyla L Davis SS. TREM2 regulates microglial cholesterol metabolism upon chronic phagocytic challenge. Neuron. (2020) 105:837–54.e9. doi: 10.1016/j.neuron.2019.12.007 31902528

[68] Jaitin DA Adlung L Thaiss CA Weiner A Li B Descamps H. Lipid-associated macrophages control metabolic homeostasis in a trem2-dependent manner. Cell. (2019) 178:686–98.e14. doi: 10.1016/j.cell.2019.05.054 31257031 PMC7068689

[69] Kohno Y Egawa Y Itoh S Nagaoka S Takahashi M Mukai K. Kinetic study of quenching reaction of singlet oxygen and scavenging reaction of free radical by squalene in n-butanol. Biochim Biophys Acta. (1995) 1256:52–6. doi: 10.1016/0005-2760(95)00005-W 7742356

[70] Ottaviani M Alestas T Flori E MastroFrancesco A Zouboulis CC Picardo M. Peroxidated squalene induces the production of inflammatory mediators in HaCaT keratinocytes: a possible role in acne vulgaris. J Invest Dermatol. (2006) 126:2430–7. doi: 10.1038/sj.jid.5700434 16778793

[71] Schönbeck U Mach F Libby P. Generation of biologically active IL-1 beta by matrix metalloproteinases: a novel caspase-1-independent pathway of IL-1 beta processing. J Immunol. (1998) 161:3340–6. 9759850

[72] Alshammrie FF Alshammari R Alharbi RM Khan FH Alshammari SK. Epidemiology of acne vulgaris and its association with lifestyle among adolescents and young adults in hail, kingdom of Saudi Arabia: A community-based study. Cureus. (2020) 12:e9277. doi: 10.7759/cureus.9277 32821620 PMC7431307

[73] Xu H Barnes GT Yang Q Tan G Yang D Chou CJ. Chronic inflammation in fat plays a crucial role in the development of obesity-related insulin resistance. J Clin Invest. (2003) 112:1821–30. doi: 10.1172/JCI200319451 PMC296998 14679177

[74] Lauterbach MA Wunderlich FT. Macrophage function in obesity-induced inflammation and insulin resistance. Eur J Physiol. (2017) 469:385–96. doi: 10.1007/s00424-017-1955-5 PMC5362664 28233125

[75] Lumeng CN Deyoung SM Bodzin JL Saltiel AR. Increased inflammatory properties of adipose tissue macrophages recruited during diet-induced obesity. Diabetes. (2007) 56:16–23. doi: 10.2337/db06-1076 17192460

[76] Weisberg SP McCann D Desai M Rosenbaum M Leibel RL Ferrante AW Jr. Obesity is associated with macrophage accumulation in adipose tissue. J Clin Invest. (2003) 112:1796–808. doi: 10.1172/JCI200319246 PMC296995 14679176

[77] Zhang LJ Guerrero-Juarez CF Chen SX Zhang X Yin M Li F. Diet-induced obesity promotes infection by impairment of the innate antimicrobial defense function of dermal adipocyte progenitors. Sci Trans Med. (2021) 13:577. doi: 10.1126/scitranslmed.abb5280 PMC8164450 33472955

[78] Chang D Xing Q Su Y Zhao X Xu W Wang X. The conserved non-coding sequences CNS6 and CNS9 control cytokine-induced rorc transcription during T helper 17 cell differentiation. Immunity. (2020) 53:614–26.e4. doi: 10.1016/j.immuni.2020.07.012 32827457

[79] Yu Y Champer J Agak GW Kao S Modlin RL Kim J. Different propionibacterium acnes phylotypes induce distinct immune responses and express unique surface and secreted proteomes. J Invest Dermatol. (2016) 136:2221–8. doi: 10.1016/j.jid.2016.06.615 PMC8295477 27377696

[80] Hong I Lee MH Na TY Zouboulis CC Lee MO. LXRalpha enhances lipid synthesis in SZ95 sebocytes. J Invest Dermatol. (2008) 128:1266–72. doi: 10.1038/sj.jid.5701134 17960176

[81] MastroFrancesco A Ottaviani M Cardinali G Flori E Briganti S Ludovici M. Pharmacological PPARγ modulation regulates sebogenesis and inflammation in SZ95 human sebocytes. Biochem Pharmacol. (2017) 138:96–106. doi: 10.1016/j.bcp.2017.04.030 28461124

[82] Melnik BC. Linking diet to acne metabolomics, inflammation, and comedogenesis: an update. Clin Cosmetic Investigational Dermatol. (2015) 8:371–88. doi: 10.2147/CCID PMC4507494 26203267

[83] Man MQ Choi EH Schmuth M Crumrine D Uchida Y Elias PM. Basis for improved permeability barrier homeostasis induced by PPAR and LXR activators: liposensors stimulate lipid synthesis, lamellar body secretion, and post-secretory lipid processing. J Invest Dermatol. (2006) 126:386–92. doi: 10.1038/sj.jid.5700046 16374473

[84] Russell LE Harrison WJ Bahta AW Zouboulis CC Burrin JM Philpott MP. Characterization of liver X receptor expression and function in human skin and the pilosebaceous unit. Exp Dermatol. (2007) 16:844–52. doi: 10.1111/j.1600-0625.2007.00612.x 17845217

[85] Feng K Ma C Liu Y Yang X Yang Z Chen Y. Encapsulation of LXR ligand by D-Nap-GFFY hydrogel enhances anti-tumorigenic actions of LXR and removes LXR-induced lipogenesis. Theranostics. (2021) 11:2634–54. doi: 10.7150/thno.53139 PMC7806465 33456564

[86] Xu HF Luo J Zhang XY Li J Bionaz M. Activation of liver X receptor promotes fatty acid synthesis in goat mammary epithelial cells via modulation of SREBP1 expression. J Dairy Sci. (2019) 102:3544–55. doi: 10.3168/jds.2018-15538 30738675

[87] Najafi-Shoushtari SH Kristo F Li Y Shioda T Cohen DE Gerszten RE. MicroRNA-33 and the SREBP host genes cooperate to control cholesterol homeostasis. Science. (2010) 328:1566–9. doi: 10.1126/science.1189123 PMC3840500 20466882

[88] Kusnadi A Park SH Yuan R Pannellini T Giannopoulou E Oliver D. The cytokine TNF promotes transcription factor SREBP activity and binding to inflammatory genes to activate macrophages and limit tissue repair. Immunity. (2019) 51:241–57.e9. doi: 10.1016/j.immuni.2019.06.005 31303399 PMC6709581

[89] Ahangari F Price NL Malik S Chioccioli M Bärnthaler T Adams TS. microRNA-33 deficiency in macrophages enhances autophagy, improves mitochondrial homeostasis, and protects against lung fibrosis. JCI Insight. (2023) 8:e158100. doi: 10.1172/jci.insight.158100 36626225 PMC9977502

[90] Lee JH Lee SH Lee EH Cho JY Song DK Lee YJ. SCAP deficiency facilitates obesity and insulin resistance through shifting adipose tissue macrophage polarization. J Adv Res. (2023) 45:1–13. doi: 10.1016/j.jare.2022.05.013 35659922 PMC10006517

[91] Trivedi NR Cong Z Nelson AM Albert AJ Rosamilia LL Sivarajah S. Peroxisome proliferator-activated receptors increase human sebum production. J Invest Dermatol. (2006) 126:2002–9. doi: 10.1038/sj.jid.5700336 16675962

[92] Chinetti G Lestavel S Bocher V Remaley AT Neve B Torra IP. PPAR-alpha and PPAR-gamma activators induce cholesterol removal from human macrophage foam cells through stimulation of the ABCA1 pathway. Nat Med. (2001) 7:53–8. doi: 10.1038/83348 11135616

[93] Downie MM Sanders DA Maier LM Stock DM Kealey T. Peroxisome proliferator-activated receptor and farnesoid X receptor ligands differentially regulate sebaceous differentiation in human sebaceous gland organ cultures *in vitro* . Br J Dermatol. (2004) 151:766–75. doi: 10.1111/j.1365-2133.2004.06171.x 15491415

[94] Rosenfield RL Kentsis A Deplewski D Ciletti N. Rat preputial sebocyte differentiation involves peroxisome proliferator-activated receptors. J Invest Dermatol. (1999) 112:226–32. doi: 10.1046/j.1523-1747.1999.00487.x 9989800

[95] Su M Cao J Huang J Liu S Im DS Yoo JW. The *in vitro* and *in vivo* anti-inflammatory effects of a phthalimide PPAR-γ Agonist. Mar Drugs. (2017) 15:7. doi: 10.3390/md15010007 28054961 PMC5295227

[96] Zhang R Zhou L Lv M Yue N Fei W Wang L. The relevant of sex hormone levels and acne grades in patients with acne vulgaris: A cross-sectional study in Beijing. Clin Cosmetic Investigational Dermatol. (2022) 15:2211–9. doi: 10.2147/CCID.S385376 PMC9587737 36281268

[97] Borzyszkowska D Niedzielska M Kozłowski M Brodowska A Przepiera A Malczyk-Matysiak K. Evaluation of hormonal factors in acne vulgaris and the course of acne vulgaris treatment with contraceptive-based therapies in young adult women. Cells. (2022) 11:4078. doi: 10.3390/cells11244078 36552842 PMC9777314

[98] Stoll S Shalita AR Webster GF Kaplan R Danesh S Penstein A. The effect of the menstrual cycle on acne. J Am Acad Dermatol. (2001) 45:957–60. doi: 10.1067/mjd.2001.117382 11712049

[99] Leis K Mazur E Jabłońska MJ Kolan M Gałązka P. Endocrine systems of the skin. Postepy Dermatol Alergol. (2019) 36:519–23. doi: 10.5114/ada.2019.89502 PMC6906956 31839767

[100] Bharti S Vadlamudi HC. A strategic review on the involvement of receptors, transcription factors, and hormones in acne pathogenesis. J Receptor Signal Transduction Res. (2021) 41:105–16. doi: 10.1080/10799893.2020.1805626 32787477

[101] Crocco EI Bonifácio EB Facchini G da Silva GH da Silva MS Pinheiro ALTA. Modulation of skin androgenesis and sebum production by a dermocosmetic formulation. J Cosmet Dermatol. (2021) 20:360–5. doi: 10.1111/jocd.13503 32433801

[102] Lv Y Chu C Liu K Ru Y Zhang Y Lu X. A combination of CMC and α-MSH inhibited ROS activated NLRP3 inflammasome in hyperosmolarity stressed HCECs and scopolamine-induced dry eye rats. Sci Rep. (2021) 11:1184. doi: 10.1038/s41598-020-80849-2 33441928 PMC7807058

[103] Lee WJ Jung HD Chi SG Kim BS Lee SJ Kim DW. Effect of dihydrotestosterone on the upregulation of inflammatory cytokines in cultured sebocytes. Arch Dermatol Res. (2010) 302:429–33. doi: 10.1007/s00403-009-1019-6 20043171

[104] Santana LCL Spolidorio LC Pitombo JCP Basso FG Guarenghi GG Prates RC. Testosterone increases fibroblast proliferation *in vitro* through androgen and estrogen receptor activation. J Int Acad Periodontol. (2020) 22:146–55. 32655040

[105] Hu T Wei Z Ju Q Chen W. Sex hormones and acne: State of the art. J Dtsch Dermatol Ges. (2021) 19:509–15. doi: 10.1111/ddg.14426 33576151

[106] Villuendas G San Millán JL Sancho J Escobar-Morreale HF. The -597 G–>A and -174 G–>C polymorphisms in the promoter of the IL-6 gene are associated with hyperandrogenism. J Clin Endocrinol Metab. (2002) 87:1134–41. doi: 10.1210/jcem.87.3.8309 11889177

[107] Romańska-Gocka K Woźniak M Kaczmarek-Skamira E Zegarska B. The possible role of diet in the pathogenesis of adult female acne. Postepy Dermatol Alergol. (2016) 33:416–20. doi: 10.5114/ada.2016.63880 PMC5183780 28035217

[108] Sadowska-Przytocka A Gruszczyńska M Ostałowska A Antosik P Czarnecka-Operacz M Adamski Z. Insulin resistance in the course of acne - literature review. Postepy Dermatol Alergol. (2022) 39:231–8. doi: 10.5114/ada.2021.107101 PMC9131965 35645675

[109] González F. Inflammation in Polycystic Ovary Syndrome: underpinning of insulin resistance and ovarian dysfunction. Steroids. (2012) 77:300–5. doi: 10.1016/j.steroids.2011.12.003 PMC3309040 22178787

[110] Su D Coudriet GM Hyun Kim D Lu Y Perdomo G Qu S. FoxO1 links insulin resistance to proinflammatory cytokine IL-1beta production in macrophages. Diabetes. (2009) 58:2624–33. doi: 10.2337/db09-0232 PMC2768186 19651810

[111] Lee S Usman TO Yamauchi J Chhetri G Wang X Coudriet GM. Myeloid FoxO1 depletion attenuates hepatic inflammation and prevents nonalcoholic steatohepatitis. J Clin Invest. (2022) 132:e154333. doi: 10.1172/JCI154333 35700043 PMC9282937

[112] Liu F Qiu H Xue M Zhang S Zhang X Xu J. MSC-secreted TGF-β regulates lipopolysaccharide-stimulated macrophage M2-like polarization via the Akt/FoxO1 pathway. Stem Cell Res Ther. (2019) 10:345. doi: 10.1186/s13287-019-1447-y 31771622 PMC6878630

[113] Fitz-Gibbon S Tomida S Chiu BH Nguyen L Du C Liu M. Propionibacterium acnes strain populations in the human skin microbiome associated with acne. J Invest Dermatol. (2013) 133:2152–60. doi: 10.1038/jid.2013.21 PMC3745799 23337890

[114] Nguyen CT Sah SK Zouboulis CC Kim TY. Inhibitory effects of superoxide dismutase 3 on Propionibacterium acnes-induced skin inflammation. Sci Rep. (2018) 8:4024. doi: 10.1038/s41598-018-22132-z 29507345 PMC5838256

[115] Barroso RA Navarro R Tim CR de Paula Ramos L de Oliveira LD Araki ÂT. Antimicrobial photodynamic therapy against Propionibacterium acnes biofilms using hypericin (Hypericum perforatum) photosensitizer: *in vitro* study. Lasers Med Sci. (2021) 36:1235–40. doi: 10.1007/s10103-020-03163-3 33083912

[116] Huang WC Tsai TH Chuang LT Li YY Zouboulis CC Tsai PJ. Anti-bacterial and anti-inflammatory properties of capric acid against Propionibacterium acnes: a comparative study with lauric acid. J Dermatol Sci. (2014) 73:232–40. doi: 10.1016/j.jdermsci.2013.10.010 24284257

[117] Dréno B Pécastaings S Corvec S Veraldi S Khammari A Roques C. *Cutibacterium acnes* (Propionibacterium acnes) and acne vulgaris: a brief look at the latest updates. J Eur Acad Dermatol Venereol. (2018) 32:5–14. doi: 10.1111/jdv.15043 29894579

[118] Kang S Cho S Chung JH Hammerberg C Fisher GJ Voorhees JJ. Inflammation and extracellular matrix degradation mediated by activated transcription factors nuclear factor-kappaB and activator protein-1 in inflammatory acne lesions *in vivo* . Am J Pathol. (2005) 166:1691–9. doi: 10.1016/S0002-9440(10)62479-0 PMC1602424 15920154

[119] Wang Y Hata TR Tong YL Kao MS Zouboulis CC Gallo RL. The anti-inflammatory activities of propionibacterium acnes CAMP factor-targeted acne vaccines. J Invest Dermatol. (2018) 138:2355–64. doi: 10.1016/j.jid.2018.05.032 29964032

[120] Shan M Meng F Tang C Zhou L Lu Z Lu Y. Surfactin-oleogel with therapeutic potential for inflammatory acne vulgaris induced by Propionibacterium acnes. Appl Microbiol Biotechnol. (2022) 106:549–62. doi: 10.1007/s00253-021-11719-8 34939137

[121] Slaby O McDowell A Brüggemann H Raz A Demir-Deviren S Freemont T. Is IL-1β Further evidence for the role of propionibacterium acnes in degenerative disc disease? Lessons from the study of the inflammatory skin condition acne vulgaris. Front Cell Infect Microbiol. (2018) 8:272. doi: 10.3389/fcimb.2018.00272 30155445 PMC6103242

[122] Novak ML Koh TJ. Macrophage phenotypes during tissue repair. J Leukocyte Biol. (2013) 93:875–81. doi: 10.1189/jlb.1012512 PMC3656331 23505314

[123] Ip WKE Hoshi N Shouval DS Snapper S Medzhitov R. Anti-inflammatory effect of IL-10 mediated by metabolic reprogramming of macrophages. Science. (2017) 356:513–9. doi: 10.1126/science.aal3535 PMC6260791 28473584

[124] Cui L Li Z Chang X Cong G Hao L. Quercetin attenuates vascular calcification by inhibiting oxidative stress and mitochondrial fission. Vasc Pharmacol. (2017) 88:21–9. doi: 10.1016/j.vph.2016.11.006 27932069

[125] Byun EB Yang MS Choi HG Sung NY Song DS Sin SJ. Quercetin negatively regulates TLR4 signaling induced by lipopolysaccharide through Tollip expression. Biochem Biophys Res Commun. (2013) 431:698–705. doi: 10.1016/j.bbrc.2013.01.056 23353651

[126] Kelly GS. Quercetin. Monograph. Altern Med Review: J Clin Therapeutic. (2011) 16:172–94. 21649459

[127] Havsteen BH. The biochemistry and medical significance of the flavonoids. Pharmacol Ther. (2002) 96:67–202. doi: 10.1016/S0163-7258(02)00298-X 12453566

[128] Lim HJ Kang SH Song YJ Jeon YD Jin JS. Inhibitory effect of quercetin on propionibacterium acnes-induced skin inflammation. Int Immunopharmacol. (2021) 96:107557. doi: 10.1016/j.intimp.2021.107557 33812252

[129] Liang R Han RM Fu LM Ai XC Zhang JP Skibsted LH. Baicalin in radical scavenging and its synergistic effect with beta-carotene in antilipoxidation. J Agric Food Chem. (2009) 57:7118–24. doi: 10.1021/jf9013263 19722585

[130] Lee W Ku SK Bae JS. Anti-inflammatory effects of Baicalin, Baicalein, and Wogonin *in vitro* and *in vivo* . Inflammation. (2015) 38:110–25. doi: 10.1007/s10753-014-0013-0 25249339

[131] Moghaddam E Teoh BT Sam SS Lani R Hassandarvish P Chik Z. Baicalin, a metabolite of baicalein with antiviral activity against dengue virus. Sci Rep. (2014) 4:5452. doi: 10.1038/srep05452 24965553 PMC4071309

[132] Fang F Xie Z Quan J Wei X Wang L Yang L. Baicalin suppresses Propionibacterium acnes-induced skin inflammation by downregulating the NF-κB/MAPK signaling pathway and inhibiting activation of NLRP3 inflammasome. Braz J Med Biol Res. (2020) 53:e9949. doi: 10.1590/1414-431x20209949 33111746 PMC7584154

[133] Zheng N Liu F Lu H Zhan Y Zhang M Guo W. Schisantherin A protects against liver ischemia-reperfusion injury via inhibition of mitogen-activated protein kinase pathway. Int Immunopharmacol. (2017) 47:28–37. doi: 10.1016/j.intimp.2017.03.019 28364626

[134] Szopa A Ekiert R Ekiert H. Current knowledge of Schisandra chinensis (Turcz.) Baill. (Chinese magnolia vine) as a medicinal plant species: a review on the bioactive components, pharmacological properties, analytical and biotechnological studies. Phytochem Rev. (2017) 16:195–218. doi: 10.1007/s11101-016-9470-4 28424569 PMC5378736

[135] Leong PK Wong HS Chen J Chan WM Leung HY Ko KM. Differential action between schisandrin A and schisandrin B in eliciting an anti-inflammatory action: the depletion of reduced glutathione and the induction of an antioxidant response. PloS One. (2016) 11:e0155879. doi: 10.1371/journal.pone.0155879 27195753 PMC4873034

[136] Guo M An F Yu H Wei X Hong M Lu Y. Comparative effects of schisandrin A, B, and C on Propionibacterium acnes-induced, NLRP3 inflammasome activation-mediated IL-1β secretion and pyroptosis. Biomed Pharmacother. (2017) 96:129–36. doi: 10.1016/j.biopha.2017.09.097 28972885

[137] Wang Y Cui J Liu M Shao Y Dong X. Schisandrin C attenuates renal damage in diabetic nephropathy by regulating macrophage polarization. Am J Trans Res. (2021) 13:210–22. PMC7847524 33527019

[138] Funakoshi-Tago M Nakamura K Tsuruya R Hatanaka M Mashino T Sonoda Y. The fixed structure of Licochalcone A by alpha, beta-unsaturated ketone is necessary for anti-inflammatory activity through the inhibition of NF-κB activation. Int Immunopharmacol. (2010) 10:562–71. doi: 10.1016/j.intimp.2010.02.003 20153843

[139] Kühnl J Roggenkamp D Gehrke SA Stäb F Wenck H Kolbe L. Licochalcone A activates Nrf2 *in vitro* and contributes to licorice extract-induced lowered cutaneous oxidative stress *in vivo* . Exp Dermatol. (2015) 24:42–7. doi: 10.1111/exd.12588 25381913

[140] Cai M Xu YC Deng B Chen JB Chen TF Zeng KF. Radix Glycyrrhizae extract and licochalcone a exert an anti-inflammatory action by direct suppression of toll like receptor 4. J Ethnopharmacol. (2023) 302:115869. doi: 10.1016/j.jep.2022.115869 36309116

[141] Kolbe L Immeyer J Batzer J Wensorra U tom Dieck K Mundt C. Anti-inflammatory efficacy of Licochalcone A: correlation of clinical potency and *in vitro* effects. Arch Dermatol Res. (2006) 298:23–30. doi: 10.1007/s00403-006-0654-4 16552540

[142] Angelova-Fischer I Rippke F Fischer TW Neufang G Zillikens D. A double-blind, randomized, vehicle-controlled efficacy assessment study of a skin care formulation for improvement of mild to moderately severe acne. J Eur Acad Dermatol Venereol. (2013) 27:6–11. doi: 10.1111/jdv.12168 23731195

[143] Yang G Lee HE Yeon SH Kang HC Cho YY Lee HS. Licochalcone A attenuates acne symptoms mediated by suppression of NLRP3 inflammasome. Phytother Res. (2018) 32:2551–9. doi: 10.1002/ptr.6195 30281174

[144] Sarwar R Farooq U Khan A Naz S Khan S Khan A. Evaluation of antioxidant, free radical scavenging, and antimicrobial activity of quercus incana roxb. Front Pharmacol. (2015) 6:277. doi: 10.3389/fphar.2015.00277 26635607 PMC4655348

[145] Kim EB Lee EK Son SY Lee MW. Antiacne and anti-inflammatory effects of phenolic compounds from quercus acutissima carruth. Leaves. Evidence-Based Complementary Altern Med. (2022) 2022:9078475. doi: 10.1155/2022/9078475 PMC9825228 36624865

[146] Kamada T Kang MC Phan CS Zanil II YJ J Vairappan CS. Bioactive cembranoids from the soft coral genus sinularia sp. in borneo. Mar Drugs. (2018) 16:99. doi: 10.3390/md16040099 29561805 PMC5923386

[147] Lin YY Jean YH Lee HP Chen WF Sun YM Su JH. A soft coral-derived compound, 11-epi-sinulariolide acetate suppresses inflammatory response and bone destruction in adjuvant-induced arthritis. PloS One. (2013) 8:e62926. doi: 10.1371/journal.pone.0062926 23675440 PMC3652811

[148] Chen LW Chung HL Wang CC Su JH Chen YJ Lee CJ. Anti-acne effects of cembrene diterpenoids from the cultured soft coral sinularia flexibilis. Mar Drugs. (2020) 18:487. doi: 10.3390/md18100487 32992719 PMC7601839

[149] He J Huang B Ban X Tian J Zhu L Wang Y. *In vitro* and *in vivo* antioxidant activity of the ethanolic extract from Meconopsis quintuplinervia. J Ethnopharmacol. (2012) 141:104–10. doi: 10.1016/j.jep.2012.02.006 22343365

[150] Gao L Xie M Zhang X Qiu Z Pu Z Huang S. Meconopsis quintuplinervia regel improves *cutibacterium acnes*-induced inflammatory responses in a mouse ear edema model and suppresses pro-inflammatory chemokine production via the mapk and nf-κb pathways in raw264. Cells Ann Dermatol. (2023) 35:408–16. doi: 10.5021/ad.22.206 PMC10733074 38086354

[151] Pitakpawasutthi Y Palanuvej C Ruangrungsi N. Quality evaluation of Kaempferia parviflora rhizome with reference to 5,7-dimethoxyflavone. J Adv Pharm Technol Res. (2018) 9:26–31. doi: 10.4103/japtr.JAPTR_147_17 29441321 PMC5801584

[152] Mekjaruskul C Jay M Sripanidkulchai B. Pharmacokinetics, bioavailability, tissue distribution, excretion, and metabolite identification of methoxyflavones in Kaempferia parviflora extract in rats. Drug Metab Disposition. (2012) 40:2342–53. doi: 10.1124/dmd.112.047142 22961680

[153] Toda K Hitoe S Takeda S Shimoda H. Black ginger extract increases physical fitness performance and muscular endurance by improving inflammation and energy metabolism. Heliyon. (2016) 2:e00115. doi: 10.1016/j.heliyon.2016.e00115 27441286 PMC4946221

[154] Potikanond S Sookkhee S Na Takuathung M Mungkornasawakul P Wikan N Smith DR. Kaempferia parviflora Extract Exhibits Anti-cancer Activity against HeLa Cervical Cancer Cells. Front Pharmacol. (2017) 8:630. doi: 10.3389/fphar.2017.00630 28955234 PMC5600991

[155] Jin S Lee MY. Kaempferia parviflora extract as a potential anti-acne agent with anti-inflammatory, sebostatic, and anti-propionibacterium acnes activity. Int J Mol Sci. (2018) 19:3457. doi: 10.3390/ijms19113457 30400322 PMC6274695

[156] Mizawa M Makino T Inami C Shimizu T. Jumihaidokuto (Shi-wei-ba-du-tang), a kampo formula, decreases the disease activity of palmoplantar pustulosis. Dermatol Res Practice. (2016) 2016:4060673. doi: 10.1155/2016/4060673 PMC4842044 27143961

[157] Higaki S Toyomoto T Morohashi M. Seijo-bofu-to, Jumi-haidoku-to, and Toki-shakuyaku-san suppress rashes and incidental symptoms in acne patients. Drugs Under Exp Clin Res. (2002) 28:193–6. 12635494

[158] Sekiguchi K Koseki J Tsuchiya K Matsubara Y Iizuka S Imamura S. Suppression of propionibacterium acnes-induced dermatitis by a traditional Japanese medicine, jumihaidokuto, modifying macrophage functions. Evidence-Based Complementary Altern Med. (2015) 2015:439258. doi: 10.1155/2015/439258 PMC4606168 26495013

[159] Zuo G Gao Y Lu G Bu M Liu J Zhang J. Auriculotherapy modulates macrophage polarization to reduce inflammatory response in a rat model of acne. Mediators Inflammation. (2023) 2023:6627393. doi: 10.1155/2023/6627393 PMC10163966 37159798

[160] Zhang Y Jiang Y Zhao J Mo Q Wang C Wang D. Weizmannia coagulans extracellular proteins reduce skin acne by inhibiting pathogenic bacteria and regulating TLR2/TRAF6-mediated NF-κB and MAPKs signaling pathways. Probiotics Antimicrobial Proteins. (2023). doi: 10.1007/s12602-023-10175-2 37870674

[161] Liu S Luo XH Liu YF Zouboulis CC Shi G. Emodin exhibits anti-acne potential by inhibiting cell growth, lipogenesis, and inflammation in human SZ95 sebocytes. Sci Rep. (2023) 13:21576. doi: 10.1038/s41598-023-48709-x 38062074 PMC10703917

[162] Mias C Mengeaud V Bessou-Touya S Duplan H. Recent advances in understanding inflammatory acne: Deciphering the relationship between *Cutibacterium acnes* and Th17 inflammatory pathway. J Eur Acad Dermatol Venereol. (2023) 37:3–11. doi: 10.1111/jdv.18794 36729400

[163] Su YT Zouboulis CC Cui W Zhang AP. Lactoferrin regulates sebogenesis and inflammation in SZ95 human sebocytes and mouse model of acne. J Cosmetic Dermatol. (2023) 22:1361–8. doi: 10.1111/jocd.15577 36700382

[164] Di H Liu H Xu S Yi N Wei G. Network pharmacology and experimental validation to explore the molecular mechanisms of compound huangbai liquid for the treatment of acne. Drug Design Development Ther. (2023) 17:39–53. doi: 10.2147/DDDT.S385208 PMC9843476 36660250

[165] Liu P Liu X Zhang L Yan G Zhang H Xu D. ALA-PDT augments intense inflammation in the treatment of acne vulgaris by COX2/TREM1 mediated M1 macrophage polarization. Biochem Pharmacol. (2023) 208:115403. doi: 10.1016/j.bcp.2022.115403 36592708

[166] Gu H An HJ Gwon MG Bae S Zouboulis CC Park KK. The effects of synthetic SREBP-1 and PPAR-γ Decoy oligodeoxynucleotide on acne-like disease *in vivo* and *in vitro* via lipogenic regulation. Biomolecules. (2022) 12:1858. doi: 10.3390/biom12121858 36551286 PMC9775059

[167] Cong TX Hao D Wen X Li XH He G Jiang X. From pathogenesis of acne vulgaris to anti-acne agents. Arch Dermatol Res. (2019) 311:337–49. doi: 10.1007/s00403-019-01908-x 30859308

[168] Harvey A Huynh TT. Inflammation and acne: putting the pieces together. J Drugs Dermatol. (2014) 13:459–63. 24719066

